# Nutrition in a lifecourse perspective: From molecular aspects to public health approaches

**DOI:** 10.1111/mcn.13582

**Published:** 2023-11-06

**Authors:** Nina C. Øverby, Elisabet R. Hillesund, Anine C. Medin, Frøydis N. Vik, Sergej M. Ostojic

**Affiliations:** ^1^ Department of Nutrition and Public Health, UiA Priority Research Centre for Lifecourse Nutrition University of Agder Kristiansand Norway

## Abstract

This Special Issue covers a variety of topics related to nutrition from a lifecourse perspective, addressing diet in sensitive periods (preconception, pregnancy and infancy/toddlerhood), in different contexts, spanning from molecular nutrition to settings and gatekeepers of diet in these sensitive periods. It highlights challenges and research gaps within the field.

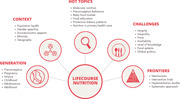

A healthy diet is fundamental to all human development and health. The most recent numbers from the Global Burden of Disease study, show that among children aged 0−9 years, the three leading detailed risk factors for attributable disability‐adjusted life years are all related to malnutrition (Murray et al., 2020). Addressing population diet is therefore at the core of health promotion and public health with huge potential to improve population health.

How can these challenges to public health be addressed? There has been increasing attention to the importance of applying a lifecourse perspective within public health, and several countries and the World Health Organization (WHO) have endorsed this approach (Public Health England, 2019; WHO Europe, 2018). The lifecourse approach takes into account that environmental exposures including biological, physical, social and behavioural factors, as well as life experiences, throughout the entire life span, influence health outcomes in current and future generations (Fine & Kotelchuck, 2010; Herman et al., 2014). Second, it acknowledges sensitive periods and ‘critical windows’ of development when environmental factors can profoundly impact long‐term health and disease risk in adulthood (Gluckman et al., 2016; Hanson & Gluckman, 2014). Preconception, pregnancy, infancy, childhood and adolescence are periods of biologic plasticity when the developing body is particularly sensitive to its surrounding environment. The lifecourse approach also acknowledges that differences in health across populations, genders, socioeconomics, caste/ethnicity, geography, and their intersections cannot be explained solely by genetics or individual choice, but rather reflect the impact of broader societal and environmental conditions over time (Fine & Kotelchuck, 2010; Herman et al., 2014).

Adequate and balanced nutrition is a cornerstone in a lifecourse approach to health, playing a pivotal biological role by providing essential energy and nutrients for developmental processes and overall well‐being. Diet, however, intertwines intricately with every facet of society, ranging from individual choices to global politics. This complexity involves drivers at structural levels such as price and availability, and individual level, such as the level of knowledge, which contribute to both inequalities and inequities.

Inequity affects people throughout the social hierarchy and is grounded in the marginalization, stigmatization or relative disempowerment of different individuals and groups. Suboptimal access to life chances (education, nutrition and more) is one of many causes of inequity (The Global Nutrition Report, 2020). According to Jones et al. (2019) a lifecourse approach leads to aetiologic insights into the developmental processes that could generate disparities in preconception, prenatally, during infancy and early childhood, through adolescence, middle adulthood, older adulthood and across generations. Thereby, embracing a lifecourse perspective in addressing nutrition holds the potential to inform the design and optimal lifecourse timing of interventions that can profoundly influence the health trajectories of individuals and populations, thereby potentially impacting intergenerational disparities.

Another hot topic within the lifecourse nutrition approach includes focusing on the family setting, encompassing both nuclear and extended families. This is where much of the cooperation regarding child rearing and nutrition care occurs (Aubel et al., 2021). In public health nutrition initiatives, the crucial role of fathers is often overlooked. It's important to pay attention to this role, as it is evolving historically and holds equal importance to the maternal role. An interesting paper from our group, published in this special issue, delves into the perspectives of both men and women on preconception, taking a step towards including fathers into such research (Valen et al., 2023).

Since its establishment in 2018, the Priority Research Centre for Lifecourse Nutrition at the University of Agder has been committed to understand and improve diet and health relations from a lifecourse perspective. Devoting this Special Issue to current advancements in the subdiscipline of lifecourse nutrition in our Centre (Bjørkkjaer et al., 2023; Helle et al., 2023; Ostojic et al., 2023; Valen et al., 2023) but also across the global research arena (Flor‐Alemany et al., 2023; Mai et al., 2023; Shinsugi & Takimoto, 2023; Thorisdottir et al., 2023) could potentially pave the path towards enhancing health outcomes for both present and future generations.

This Special Issue covers a variety of topics related to nutrition from a lifecourse perspective, addressing diet in sensitive periods (preconception, pregnancy and infancy/toddlerhood), and spanning from molecular nutrition to food environments and settings and gatekeepers of diet in these sensitive periods.

The importance of diet in the preconception years has increasingly been acknowledged the last 10 years. Ostojic et al. (2023) summarize the current evidence of the molecular perspective during preconception and next‐generation health, outlining the main metabolic networks involved in nutritional biology of this sensitive period. Valen et al. (2023) address the preconception phase on a more behavioural notion, by exploring young adults' awareness of preconception nutritional health. Using a grab sample method, short interviews with 33 men and women aged 18−45 years form the basis of their results. Their findings indicate that young adults possess some basic knowledge of healthy behaviours during pregnancy but are generally unaware of the importance of the preconception health and the role of nutrition in this phase. This highlights a need of including this topic in public education and health care.

Bjørkkjær et al. (2023) bring forward an important aspect in the aim of improving child and lifecourse diet on a structural level. They use the Norwegian Food and Health subject, which is obligatory in Norwegian schools, as a case to discuss how to use this subject to promote diet‐related life skills. They specifically focus on formal teacher qualification and more time for student reflections on the topics to improve its potential.

Also on a structural level, Thorisdottir et al. address the early part of the lifecourse by describing temporal changes in commercial baby food (CBF) market. Baby foods are widely available in food retail establishments, but often monotonous in taste and texture and of differing nutritional quality (Thorisdottir et al., 2023). In line with suggestions from the WHO, the authors present data collected in Iceland from 2016, 2019 and 2021. They report reduced availability of CBF for the age group 4−11 months over these years, but an increase in products intended from 12 months+, driven by quadrupling the assortment of so called ‘finger foods’ (ready‐to eat dry pieces of food typically to be unpacked and given to child for self‐feeding, predominantly sweet). In conclusion, the authors are concerned about the availability of high sugar food directed to children in Iceland and suggest stronger national regulations regarding the promotion of such foods.

Flor‐Alemany et al. (2023) address pregnancy in the Spanish study GESTAFIT: Dysregulation of inflammatory and cardiometabolic markers may lead to a higher risk of developing pregnancy‐related complications such as preeclampsia and gestational diabetes mellitus. Maternal dietary choice is a potentially modifiable behaviour that might positively impact maternofoetal immunometabolic markers. Results from the GESTAFIT project including 152 women, adds to the knowledge base by showing that adherence to the Mediterranean diet during pregnancy was associated with better maternal lipid serum markers throughout pregnancy.

Shinsugi and Takimoto (2023) present findings from the National Growth Survey in Japan, examining factors associated with physical growth status among children aged 12−59 months. The study results suggest that maternal health conditions, such as prepregnancy body mass index, breastfeeding and complementary feeding status (and more) are associated with physical growth in Japanese young children. These are important findings as there is little attention in policy and research in Japan regarding environmental factors relevant for growth.

Focusing on older age groups (9−11 years), Mai et al. (2023) explored dietary patterns and child, parental and societal factors associated with overweight and obesity in Vietnamese children. Nearly 60% of the 9 to 11‐year‐olds had overweight or obesity in the researched city, highlighting the importance of understanding factors associated with this phenomenon. The authors found that children with higher scores on discretionary food intake had higher odds of being overweight. They also describe a shift in the association between socioeconomic status and obesity, showing that while previous evidence has reported children living in high‐income families to be more obese, their findings show the opposite, that those living in low‐income families are more obese.

Lastly, Helle et al. (2023) provide new and valuable insights into how public health nurses (PHN) work with early life nutrition in primary health care. This is one of the first papers describing this in a Norwegian setting. Results from interviews showed that guidance on diet and nutrition is perceived by PHN as a core activity in their communication with parents of infants and toddlers. They do, however, strive to tailor their counselling to individual needs. The authors emphasize that there seems to be a discrepancy between how nutrition is prioritized in the education of PHN and the challenges they encounter in clinical practice.

A lifecourse perspective to nutrition and its relation to long‐term health considers the critical stages, transitions and settings where large differences can be made in the promotion of health and well‐being. Still, research and policy wise, there are several gaps in the literature and in policy documents. The lifecourse approach has been predominantly focused on epidemiology. It's crucial to broaden our understanding to encompass biological pathways and develop interventions that enhance diet during pivotal stages of development, all of which have long‐term impacts. Researchers and policy makers should acknowledge and take into account the importance of early interventions in relation to, for example, noncommunicable diseases. There is also a call for implementing efficacious interventions at scale, while actively seeking to include user voices and voices of those seldom heard. Interventions should take a systems approach, addressing both individual and structural levels, as highlighted in this issue where approaches to food retailers, schools and health care are presented (Bjørkkjær et al., 2023; Helle et al., 2023; Thorisdottir et al., 2023). As commented in the Global Nutrition Report (2020), there is also a call for nutrition equity sensitive policies. Inequalities and inequities in nutrition are discussed in several papers in this supplement (Mai et al., 2023; Shinsugi & Takimoto, 2023).

This Special Issue presents eight papers with authors from 11 different countries (Australia, Austria, Finland, Hungary, Iceland, Japan, Norway, Spain, Sweden, United Kingdom and Vietnam), totalling 32 unique authors, highlighting the global lifecourse approach to nutrition. The supplement we edited provides important insights related to different phases, different arenas and different levels of action, and provides suggestions for the way forward to make a difference for better lifecourse nutrition and health.
